# Thyroid hormone receptor α signaling shapes innate and adaptive immune responses during viral infection

**DOI:** 10.1530/ETJ-25-0156

**Published:** 2025-11-06

**Authors:** Christina Wenzek, Torben Knuschke, G Sebastian Hönes, Anita Boelen, Robert Klopfleisch, Denise Zwanziger, Heike Heuer, Astrid M Westendorf, Lars C Moeller, Dagmar Führer

**Affiliations:** ^1^ Department of Endocrinology, Diabetes and Metabolism, University Hospital Essen, University Duisburg-Essen, Essen, Germany; ^2^ Institute of Medical Microbiology, University Hospital Essen, University Duisburg-Essen, Essen, Germany; ^3^ Endocrine Laboratory, Department of Laboratory Medicine, Amsterdam UMC, University of Amsterdam, Research Institute Amsterdam Gastroenterology Endocrinology and Metabolism, Amsterdam, Netherlands; ^4^ Institute of Veterinary Pathology, Freie Universität Berlin, Berlin, Germany

**Keywords:** thyroid hormone, thyroid hormone receptor α, influenza virus infection, anti-viral immunity

## Abstract

**Objective:**

Thyroid hormones (TH) are well-known regulators of the immune system. However, the precise immunomodulatory mechanisms of TH action in immune cells remain elusive. In a previous study, an essential role of the TH receptor α (TRα) in regulatory T cell (Treg) immunity was demonstrated, affecting Treg activation at steady state. The present study therefore aimed to unravel the biological relevance of altered TRα action in protective immune responses during disease.

**Methods:**

To assess the role of TRα action in immune responses, especially T cell responses, during disease, different TRα signaling mouse models (TRαKO, complete lack of TRα signaling; TRαGS, lack of canonical signaling) were challenged with influenza virus A/PR8/34, and in-depth immune phenotyping was performed.

**Results:**

Upon influenza virus infection, TRαGS mice, which lack canonical TRα signaling, showed prolonged survival and reduced disease severity, correlating with enhanced anti-inflammatory Treg and decreased pro-inflammatory CD4 and CD8 T cell responses. The loss of TRα action in TRαKO mice was related to elevated viral titers upon influenza virus infection, which correlated with increased inflammatory monocyte responses early during infection.

**Conclusion:**

The present study demonstrates a complex role of TRα signaling in protective immune responses during disease, with distinct effects on innate and adaptive immune cells. By exploring the understudied link between the endocrine and immune systems, this study provides novel evidence for the role of TH as modulators of immunity.

## Introduction

Thyroid hormones (TH) are well-known regulators of the immune system, controlling innate and adaptive immune cell function (1). Within innate immune cells, TH were primarily described to promote pro-inflammatory activation, whereas the immunomodulatory function of TH in adaptive immune cells, such as conventional T cells, remains largely elusive.

Intracellular signaling of TH in target cells is mediated by TH receptors (TRs), TRα and TRβ, which implement two modes of action (2). The canonical action of TH via nuclear TRs regulates target gene expression, while the noncanonical action activates cytosolic signaling pathways (3, 4). In a previous study, an in-depth characterization of mice lacking TRα (TRαKO) or TRβ (TRβKO), or carrying a mutation in the DNA-binding domain of TRα (TRαGS) or TRβ (TRβGS), was performed. This study demonstrated an essential role of TRα in CD4 T cell homeostasis in steady state (3, 5). The impaired DNA-binding of TRα in TRαGS mice abrogates canonical TRα action, while noncanonical TRα signaling is preserved. In these mice, regulatory T cells (Treg) and the activated Treg phenotype were increased, with a potential role of NF-κB signaling (6). Still, the biological relevance of these changes and the role of TRα signaling in protective immune responses during disease remain to be elucidated.

This study shows a significant effect of altered TRα signaling on disease severity and survival during acute influenza A virus (IAV) infection. In line with previous findings, TRαGS mice exhibited enhanced anti-inflammatory Treg activation, correlating with decreased inflammation mediated by cytotoxic CD8 T cells and pro-inflammatory CD4 T cell responses. On the contrary, complete loss of TRα signaling was associated with elevated viral titers at peak and augmented inflammatory monocyte responses early during infection. These results demonstrate an important role of TRα signaling in immune responses during viral infection, with differential effects on innate and adaptive immunity.

## Materials and methods

### Mice

All mouse strains used were bred and housed under specific pathogen-free (SPF) conditions in the local animal facility of the University Hospital Essen. Mice were housed at 21 ± 1°C under a 12 h light:12 h darkness cycle. Standard chow and tap water were provided *ad libitum*. Female, homozygous TRα0/0 (Thra^tm2Jas^ here to referred as TRαKO) mice, TRα^GS/GS^ (Thra^tm1Lmoe^ here referred to as TRαGS) mice, and corresponding wild-type littermates (TRαWT) were studied at an age of 12–18 weeks (3). Both mouse strains are on a C57BL/6J background. Before infection studies, TRαGS and TRαKO mouse strains were co-housed to allow harmonization of the microbiome, to exclude an impact of the microbiome on the immune response during infection.

### *In vivo* IAV infection model

Infection studies were performed using the influenza virus A/PR/8/34 (H1N1) strain, as previously described (7). Animal experiments were performed in strict accordance with the German regulations of the Society for Laboratory Animal Science (GV-SOLAS) and the European Health Law of the Federation of Laboratory Animal Science Associations (FELASA). The experimental protocols were approved by the North Rhine-Westphalia State Agency for Nature, Environment, and Consumer Protection (LANUV). During infectious studies, mice were housed in the infection unit of our animal facility, which allows exceptions from SPF FELASA recommendations.

The severity of lung pathology was scored by histopathology, as previously described (7). Apoptosis was examined in lung sections by terminal deoxynucleotidyl transferase dUTP nick-end labeling (TUNEL). Serum TH concentrations were quantified by UPLC-MS/MS (8), with minor adaptations to the sample pretreatment, as previously described (9). Cytokines were measured in serum and supernatants from lung tissue using the Luminex assay (Bio-Techne, R&D Systems, Germany). Immune responses in lung and lymph nodes were analyzed at indicated time points by flow cytometry. Single cells for analysis were obtained as previously described (7). For a detailed description, see the Supplement (see section on Supplementary materials given at the end of the article).

### Flow cytometry

Isolated leukocytes were stained using marker-specific fluorochrome-labeled antibodies and fixable viability dye eFluor780 (65-0865-18, eBioscience, Germany). *In vitro* activation and proliferation assays of isolated cells were performed as previously described (6). To analyze intracellular cytokines, cells were stimulated for 4 h with 1 μg/mL ionomycin (10634, Sigma-Aldrich, Germany), 10 ng/mL phorbol 12-myristate 13-acetate (PMA) (P1585, Sigma-Aldrich, Germany), and 5 μg/mL brefeldin A (B7561, Sigma-Aldrich, Germany), and intracellular staining was performed with the Foxp3 Transcription Factor Staining Buffer Kit (00-5523-00, eBioscience, Germany).

Samples were acquired using the LSRFortessa Cell Analyzer (BD Biosciences, Germany) and analyzed using FlowJo^TM^ version 10.8 software (BD Biosciences, Germany).

### Gene-expression analysis

Lungs were perfused with PBS, dissected, and homogenized in PBS with 0.03% BSA. Total RNA was isolated from lung supernatant (RNeasy Kit; Qiagen, Germany) and stored at −80°C. cDNA synthesis and qRT-PCR analysis were performed as previously described (6, 10). For a detailed description, see the Supplement.

### Statistical analysis

The ROUT method was used to identify outliers (11), and the D′ Agostino and Pearson omnibus normality test was performed to test for normal distribution. Means of TRαKO and TRαGS mice were compared to TRαWT by the Kruskal–Wallis test with Dunn’s correction, or one-way ANOVA with Dunnett’s correction. Statistics for two or more groups with multiple variants were done by two-way ANOVA with Dunnett’s correction. Statistical analyses were performed using GraphPad Prism v. 10.4.0 for Windows (GraphPad Software, USA). For all statistical analyses: *P < 0.05; **P < 0.01; ***P < 0.001; ****P < 0.0001.

## Results

### Abnormal pulmonary T cell phenotype in mice lacking canonical TRα signaling only

**Figure 1:**
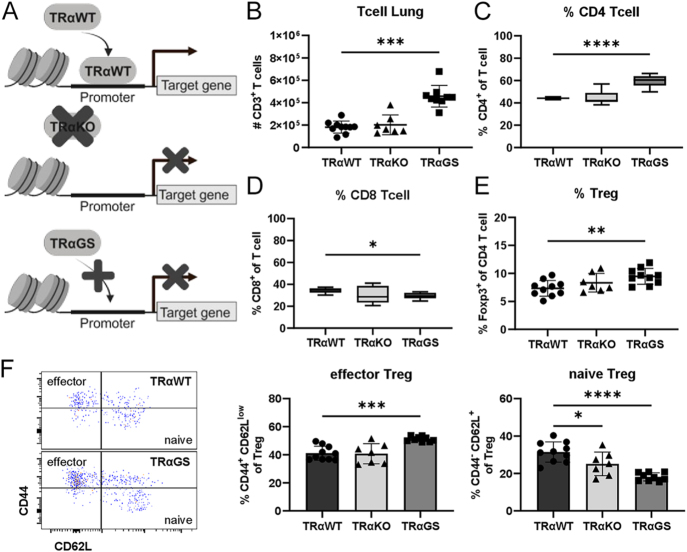
Abnormal pulmonary T cell phenotype in mice lacking canonical TRα signaling only. (A) Schematic view of utilized models comprising TRαWT mice, TRαKO mice lacking TRα, and TRαGS mice expressing mutant TRα incapable of DNA binding. (B) Total T cell number, as well as frequencies of (C) CD4 and (D) CD8 T cell subsets, were determined by flow cytometry in naïve mice. (E) Proportion of Foxp3+ regulatory CD4 T cells (Treg). (F) Effector and naïve Treg were identified as CD44+ CD62L low and CD44- CD62L high, respectively. Data are shown as mean ± SD (B, E, F) or as median ± 5–95 percentile (C and D) pooled from three independent experiments (*n* = 7–11). *P < 0.05; **P < 0.01; ***P < 0.001; ****P < 0.0001 by Kruskal–Wallis test (B) or by one-way ANOVA (C, D, E, F).

To examine the biological relevance of TRα signaling during acute respiratory disease, the phenotype of pulmonary T cell populations in uninfected mice was addressed initially. Complete lack of TRα signaling (TRαKO) had no effect, while the number of T cells in the lung was elevated in TRαGS mice, which solely lack canonical TRα action, compared to TRαWT mice (Fig. 1A and B) (6). This increase in T cells was related to a shift in the CD4/CD8 T cell ratio due to an elevated abundance of total CD4 T cells (Fig. 1C and D). Moreover, Treg frequencies, a specialized subset of CD4 T cells, were augmented in the lungs of TRαGS mice (Fig. 1E), showing an enhanced CD44^+^ CD62L^low^ effector phenotype compared to TRαWT Treg (Fig. 1F).

### Altered TRα signaling attenuates the severity of IAV infection

**Figure 2:**
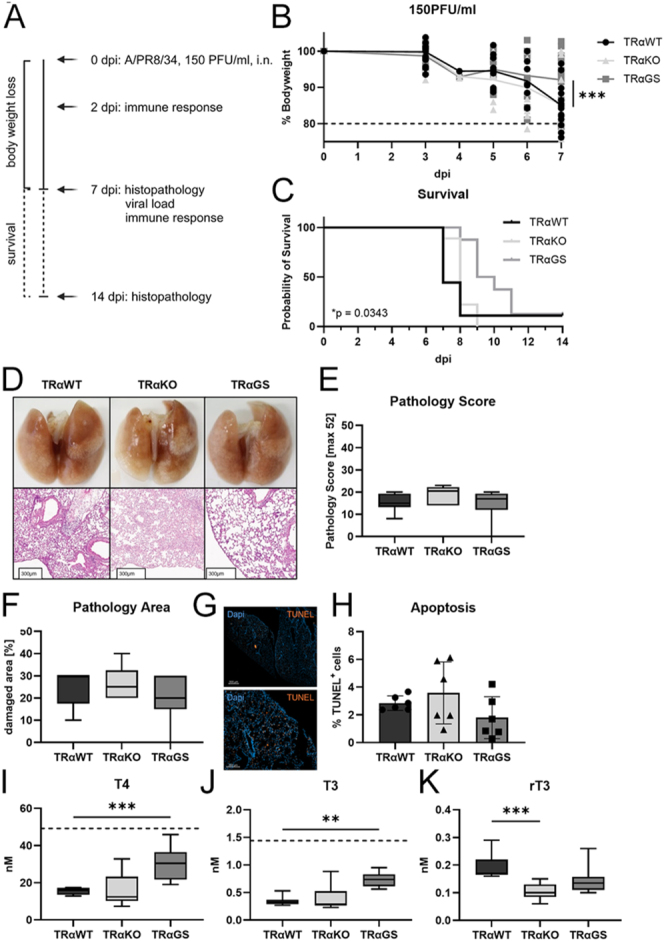
Altered TRα signaling attenuates the severity of IAV infection. (A) IAV infection model was used to challenge female TRαWT, TRαKO, and TRαGS mice. (B) The course of disease was monitored based on body weight loss, and (C) survival was determined upon low-dose infection (150PGU/mL). (D) Exemplary macroscopic and microscopic pictures of infected lung tissue. (E) Degree of tissue damage and (F) the affected area were scored. (G) Apoptosis was assessed in infected lung tissue by TUNEL staining, and (H) the ratio of apoptotic cells of total cells (Dapi) was determined. (I) Total T4 and (J) T3 levels were measured by LCMS in serum of infected mice 7 days post infection (dpi). Dashed line indicates mean concentration in healthy mice. (K) Likewise, reverse T3 (rT3) concentrations were determined. Data are shown as mean ± SD (B and H) or as median ± 5–95 percentile (E, F, I, J, K) pooled from three independent experiments (*n* = 6–9). **P < 0.01; ***P < 0.001 by two-way ANOVA (B), by log-rank (Mantel-Cox) test (C), by Kruskal–Wallis test (E, F, H, J, K), or by one-way ANOVA (I).

Next, mice were challenged with a low dose (150 PFU/mL) intranasal influenza virus A/PR8/34 infection (Fig. 2A). At the peak of infection, 7 days post infection (dpi), body weight loss was significantly attenuated in TRαGS compared to TRαWT mice (Fig. 2B). Accordingly, TRαGS mice showed prolonged survival (Fig. 2C). The severity and area of tissue pathology, as well as the ratio of apoptotic cells in the lung remained unchanged at 7 dpi (Fig. 2D, E, F, G, H). As circulating TH concentrations were demonstrated to correlate with disease severity (12, 13), serum TH concentrations were examined. Serum T4 and T3 concentrations dropped in IAV-infected TRαWT and TRαKO mice at 7 dpi, whereas TRαGS mice displayed significantly higher levels of T4 and T3 (Fig. 2I and J). Furthermore, an increased concentration of rT3 was detected in TRαWT compared to TRαKO and TRαGS mice during infection (Fig. 2K).

Increasing the initial infectious dose to medium (300/PFU/mL) or high (600/PFU/mL) levels reduced the impact of TRα on disease severity. Body weight loss of TRαGS mice gradually worsened, resembling the course of disease in TRαWT mice at high dose (Supplemental Fig. 1A and B). In line with this, tissue pathology was equally pronounced in TRαGS compared to TRαWT mice (Supplemental Fig. 1C and F).

### TRαGS mice show impaired CD8 T cell responses during IAV infection

The clearance of IAV during infection is dependent on conventional T cells. Cytotoxic CD8 T cells induce apoptosis of infected cells to limit viral replication and propagation (14, 15).

**Figure 3:**
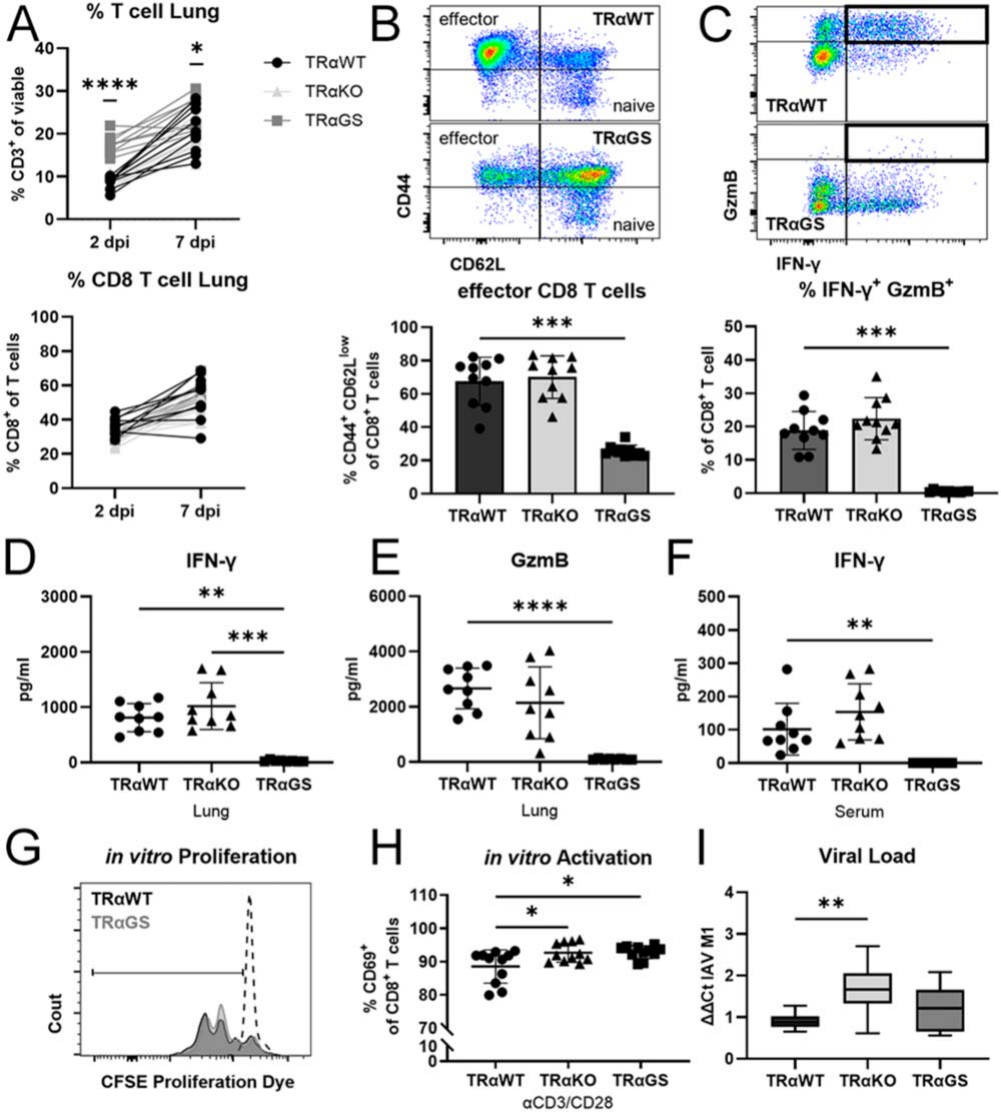
TRαGS mice show impaired CD8 T cell responses during IAV infection. (A) The frequency of T cells and CD8 T cell subset was determined by flow cytometry 2 and 7 days post infection (dpi). (B) Moreover, the proportion of CD44+ CD62L low effector CD8 T cells and (C) interferon-γ (IFN-γ)+ and granzyme B (GzmB)+ cytotoxic CD8 T cells was assessed 7 dpi. (D) Concentration of IFN-γ and (E) GzmB in lung, as well as (F) IFN-γ levels in serum, were measured by Luminex 7 dpi. (G) Exemplary graph showing proliferation of isolated CD8 T cells upon *in vitro* activation (unstimulated control = dashed line), and (H) ratio of activation marker CD69+ CD8 T cells. (I) Viral load was determined in lung tissue based on expression of viral matrix protein 1 (M1). Data are shown as mean ± SD (A, B, C, D, E, F, H) or as median ± 5–95 percentile (I) pooled from three independent experiments (*n* = 8–11). *P < 0.05; **P < 0.01; ***P < 0.001; ****P < 0.0001 by two-way ANOVA (A), by Kruskal–Wallis test (B, C, D, F), or by one-way ANOVA (E, H, I).

As observed in the healthy state, the proportion of T cells in the lung was elevated in IAV-infected TRαGS compared to TRαWT mice. Yet, the CD8 T cell subset was equally recruited during infection (Fig. 3A). The activation of CD8 T cells was markedly abated in TRαGS mice at 7 dpi. Reduced frequencies of CD44^+^ CD62L^low^ effector CD8 T cells were found in the lungs of TRαGS mice compared to TRαWT mice (Fig. 3B). The ratios of effector cytokine interferon-γ (IFN-γ) and granzyme B (GzmB) producing cytotoxic CD8 T cells were also diminished. In line with this, concentrations of IFN-γ and GzmB in the lung (Fig. 3C, D, E) and serum concentrations of IFN-γ were significantly reduced (Fig. 3F). Upon *in vitro* stimulation, equal proliferation and elevated levels of activation marker CD69 were detected in TRαGS CD8 T cells compared to TRαWT cells (Fig. 3G and H).

Despite decreased CD8 T cell responses in IAV-infected TRαGS mice, viral load was not affected at 7 dpi (Fig. 3I). Yet, viral titers were elevated in the lungs of TRαKO compared to TRαWT mice.

### CD4 T helper cell responses are inhibited in TRαGS mice during IAV infection

Besides CD8 T cells, CD4 T cells play an important role in antiviral immunity, as T helper (Th) cells, e.g., promote proper CD8 T cell responses (16, 17).

**Figure 4:**
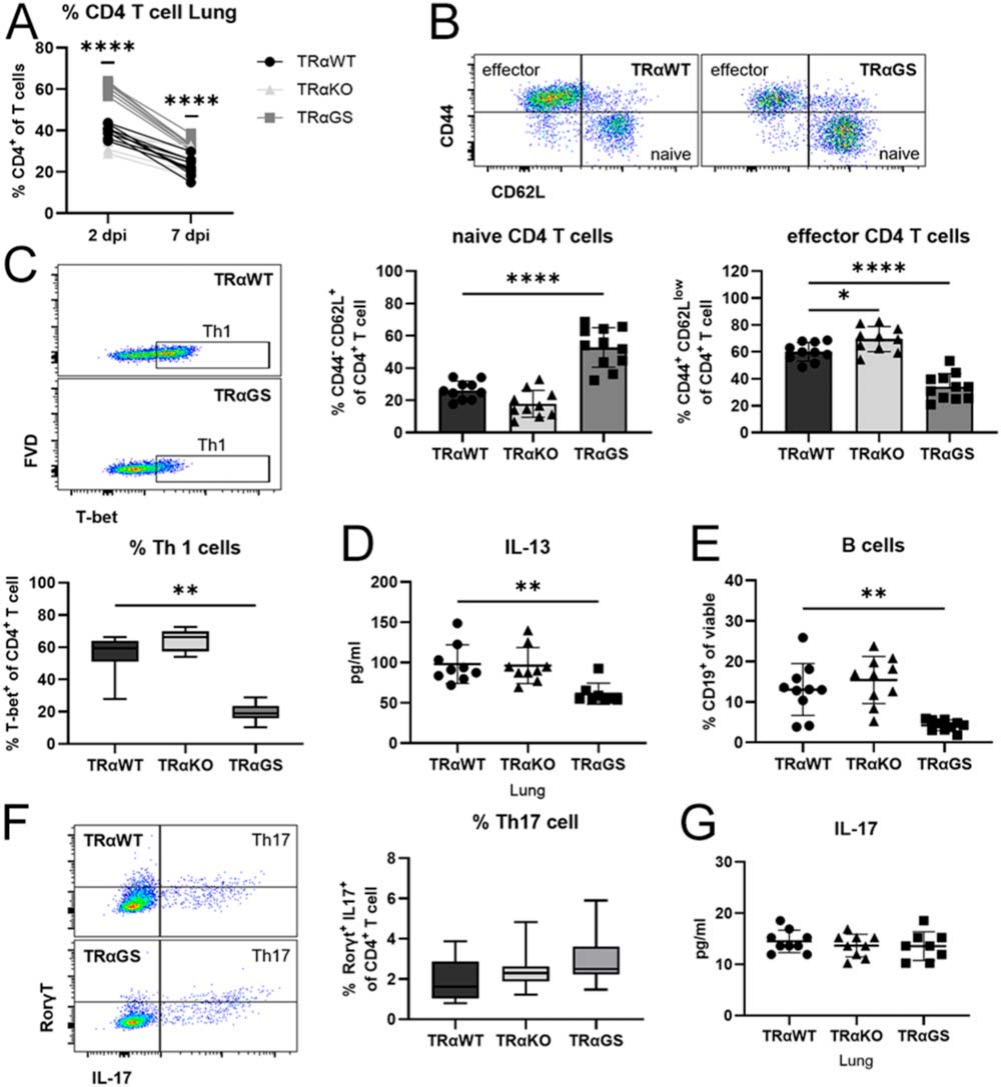
CD4 Th cell responses are inhibited in TRαGS mice during IAV infection. (A) CD4 T cell frequencies were determined by flow cytometry 2 and 7 days post infection (dpi). (B) Naïve and effector CD4 T cells were identified as CD44- CD62L high and CD44+ CD62L low, respectively. (C) Frequency of T-bet + T helper 1 (Th1) cells was assessed 7 dpi. (D) Concentration of Th2 cytokine IL-13 was determined in lung by Luminex, and (E) B cell frequencies were determined by flow cytometry. (F) Likewise, the proportion of Rorγt + IL-17+ Th17 cells was analyzed, and (G) IL-17 concentration in lung was measured by Luminex. Data are shown as mean ± SD (A, B, D, E, G) or as median ± 5–95 percentile (C and F) pooled from three independent experiments (*n* = 8–11). *P < 0.05; **P < 0.01; ****P < 0.0001 by two-way ANOVA (A), by one-way ANOVA (B, E, G), or by Kruskal–Wallis test (C, D, F).

An increased proportion of the CD4 T cell subset was detected in TRαGS mice during IAV infection (Fig. 4A). The phenotype of CD4 T cells was significantly shifted from effector to naïve phenotype (Fig. 4B). Diminished Th1 frequencies were found in TRαGS mice, which promote CD8 T cell activation during IAV infection (Fig. 4C). Moreover, a reduced concentration of IL-13, a cytokine related to Th2 cells, which promote humoral immunity via B cells, was detected in the lungs of IAV-infected TRαGS mice (Fig. 4D) (18). In line with this, decreased B cell frequencies were observed (Fig. 4E). As a former study observed an impact on Th17 polarization of TRαGS T cells *in vitro*, Th17 responses in IAV-infected mice were analyzed in addition to major Th subsets (6). During IAV infection, no differences in Th17 cells and effector cytokine IL-17 were detected in TRαGS compared to TRαWT mice (Fig. 4F and G).

### Attenuated TRαGS T cell activation is not related to impaired T cell priming by dendritic cells in lymph nodes

The induction of antiviral T cell responses is facilitated by antigen-presenting cells, such as dendritic cells (DCs), in lymph nodes. These cells present viral antigens to the T cell receptor via major histocompatibility complex (MHC) molecules.

**Figure 5:**
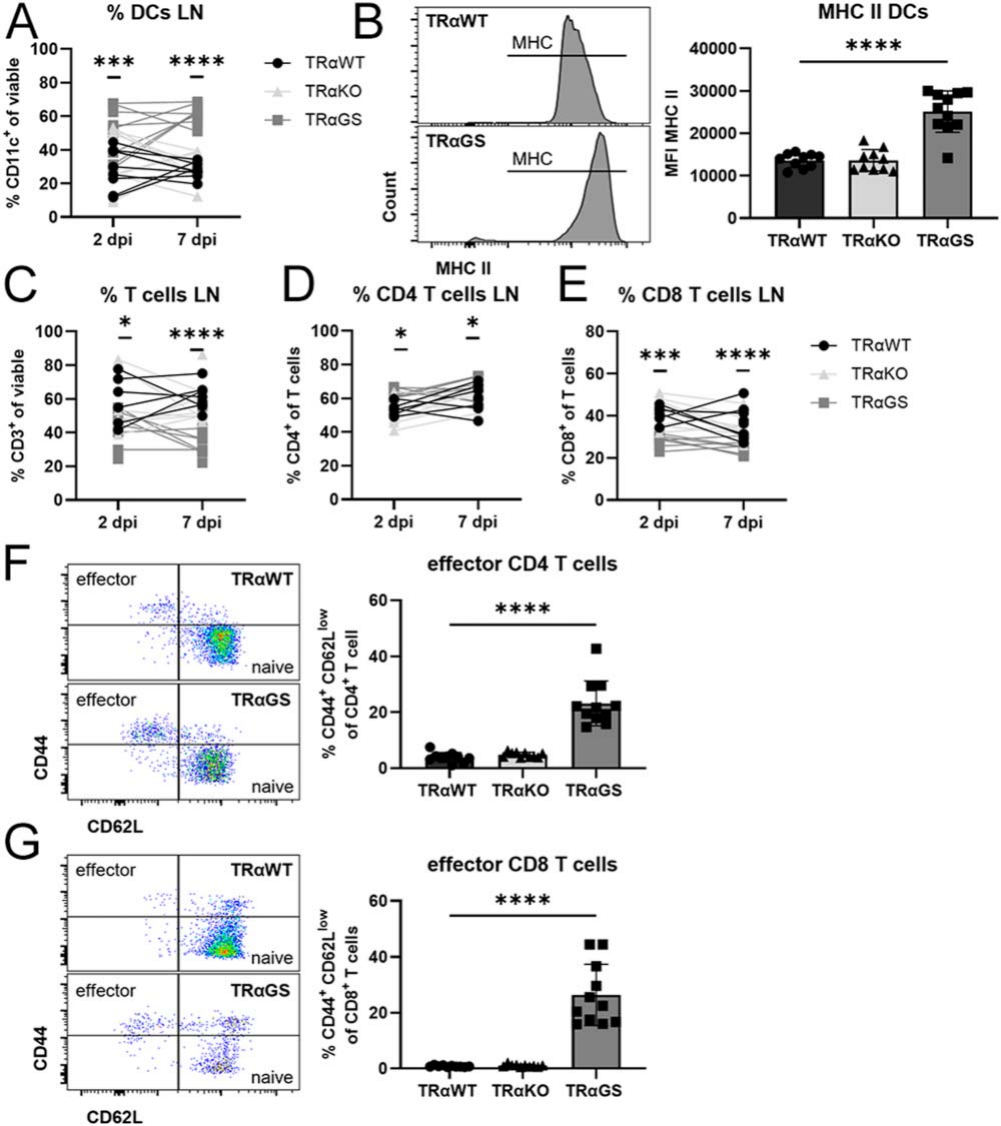
T cell priming by dendritic cells in lymph nodes is not impaired in TRαGS mice during infection. (A) Frequency of dendritic cells (DCs), and (B) protein levels of MHC class II on DCs (7 dpi) in lymph nodes of IAV-infected mice were analyzed by flow cytometry. (C) Frequency of T cells, and (D) CD4 and (E) CD8 T cell subsets in lymph nodes were assessed, and (F) effector CD4 and (G) CD8 T cells were identified as CD44+ CD62L low. Data are shown as mean ± SD pooled from three independent experiments (*n* = 8–11). *P < 0.05; ***P < 0.001; ****P < 0.0001 by two-way ANOVA (A, C, D, E), by one-way ANOVA (B and G), or by Kruskal–Wallis test (F).

To examine the role of DCs and T cell priming in the observed T cell phenotype, DC responses in lymph nodes were determined by flow cytometry. Frequencies of DCs were elevated in the lymph nodes of TRαGS mice compared to TRαWT mice early and at the peak of infection (Fig. 5A). Moreover, MHC class II was increased on DCs in the lymph nodes of TRαGS mice (Fig. 5B). Despite this increase, reduced T cell frequencies were detected in the lymph nodes of IAV-infected TRαGS mice. The CD4/CD8 T cell ratio was shifted due to a rise in CD4 T cells, while the CD8 T cell subset was reduced (Fig. 5C, D, E). Yet, in line with augmented DC responses, activation of CD4 and CD8 T cells in lymph nodes was enhanced in TRαGS mice, which show augmented frequencies of CD4 and CD8 effector T cells (Fig. 5F and G).

### Enhanced pro-inflammatory monocytes in the absence of TRα signaling

**Figure 6:**
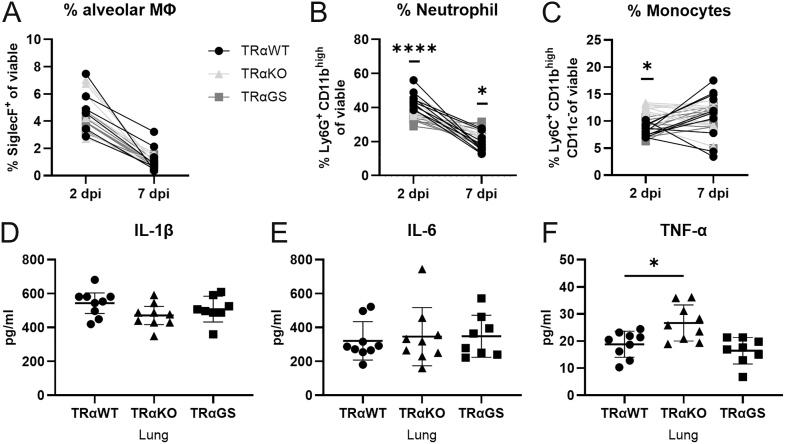
TRα signaling affects pro-inflammatory neutrophil and monocyte responses during IAV infection. (A) Frequency of alveolar macrophages, (B) neutrophils, and (C) monocytes was determined by flow cytometry in lungs of infected mice. (D) Concentration of IL-1β, (E) IL-6, and (F) TNF-α in lungs was measured by Luminex 7 dpi. Data are shown as mean ± SD pooled from three independent experiments (*n* = 8–11). *P < 0.05; ****P < 0.0001 by two-way ANOVA (A, B, C), by one-way ANOVA (D and F), or by Kruskal–Wallis test (E).

Besides T cell priming, innate immune responses at the site of infection were shown to affect T cell recruitment and expansion (19, 20, 21). Therefore, major innate immune subsets were examined in the lungs of IAV-infected mice early and at the peak of infection by flow cytometry. No differences in alveolar macrophages were observed, which represent the first line of defense in pulmonary infections (Fig. 6A) (7, 22). The proportion of neutrophils recruited upon infection was diminished early during infection in TRαGS compared to TRαWT mice (Fig. 6B) (23). However, persistent neutrophil invasion was detected in the lungs of TRαGS mice during infection. Loss of TRα in TRαKO mice significantly affected the monocyte response, leading to an increased abundance of monocytes early during infection (Fig. 6C). Furthermore, an increased concentration of the pro-inflammatory cytokine TNF-α was found in the lungs of TRαKO mice at the peak of infection, whereas other cytokines, e.g. IL-1β and IL-6, were not altered (Fig. 6D, E, F).

### Augmented regulatory T cell responses in IAV-infected mice in the absence of canonical action

**Figure 7:**
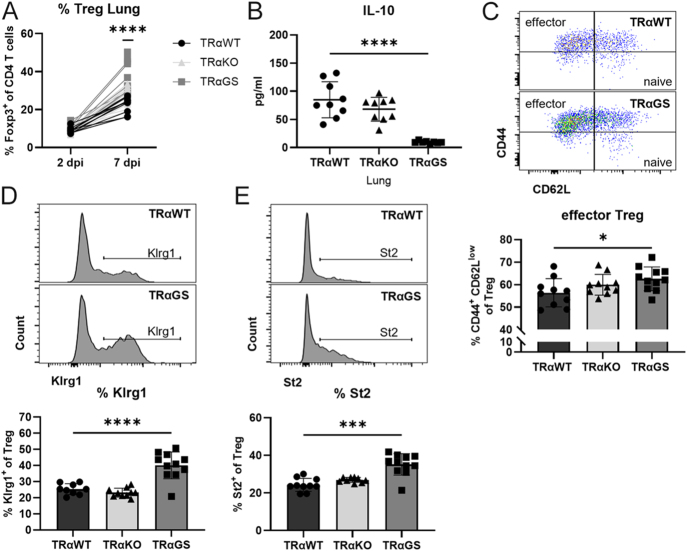
Augmented regulatory T cell responses in IAV-infected mice in the absence of canonical TRα action. (A) Frequency of pulmonary Foxp3+ regulatory T cells (Treg), (B) CD44+ CD62L low effector Treg, (C) Klrg1+ Treg, and (D) St2+ Treg were determined 7 dpi by flow cytometry. (E) Concentration of IL-10 in lungs was examined by Luminex. Data are shown as mean ± SD pooled from three independent experiments (*n* = 8–11). *P < 0.05; ***P < 0.001; ****P < 0.0001 by two-way ANOVA (A), by one-way ANOVA (B, C, D), or by Kruskal–Wallis test (E).

Given the increased frequency and activation of Treg observed in naïve TRαGS mice (Fig. 1E and F), subsequent analyses focused on Treg responses during IAV infection as a potential mechanism limiting pro-inflammatory T cell immunity. Treg frequencies and activation in lung tissue of IAV-infected mice were assessed by flow cytometry. Augmented Treg frequencies were found in lungs of TRαGS mice at the peak of infection (Fig. 7A). Despite reduced concentrations of anti-inflammatory IL-10 in the lungs of TRαGS mice, TRαGS Treg displayed an enhanced CD44^+^ CD62L^low^ effector phenotype compared to TRαWT Treg (Fig. 7B and C). Moreover, increased surface expression of functional markers Klrg1 and St2 was detected on TRαGS Treg (Fig. 7D and E).

## Discussion

TH are known regulators of innate and adaptive immune cell function; however, comprehensive understanding of the precise impact of TH/TR action, especially in adaptive immune cells, is unknown. A preceding study demonstrated a significant effect of TRα signaling on T cells and Treg in blood and spleen of healthy mice (6). Performing in-depth immune phenotyping of healthy mice, the study revealed increased CD4 T cell frequencies in mice that lack canonical TRα action (TRαGS) only. *In vitro* polarization of isolated CD4 T cells showed no differences in pro-inflammatory Th1 differentiation of TRαGS and TRαWT T cells. In line with this, RNA sequencing of isolated CD25- CD4+ naive T cells of TRαGS and TRαWT mice demonstrated differential expression of genes associated with lymphocyte homeostasis and apoptosis, indicating no major impact on pro-inflammatory CD4 T cell immunity. Instead, increased abundance of anti-inflammatory Treg was found in the spleen of TRαGS mice. Elevated frequencies of thymic Treg and enhanced differentiation of TRαGS CD4 T cells into induced Treg *in vitro* implied an important role of TRα action in Treg differentiation. Further RNA sequencing analysis of isolated CD25+ CD4+ Treg from spleen showed that genes related to T cell activation, differentiation, and migration were enriched in TRαGS Treg (e.g. *S100a4*, *Rora*, *Gzmb*, and *Il1rl1* (St2)). Together with an enhanced effector phenotype (CD62L^low^ CD44+) of TRαGS Treg and augmented levels of Treg functional markers (e.g. Icos, Klrg-1, St2), these findings suggest an important role of TRα signaling in Treg activation and function (6).

Using the TRαGS mouse model and mice that completely lack TRα (TRαKO), the present study shows a shift in CD4/CD8 subset ratio in pulmonary T cell populations of TRαGS mice. Moreover, a rise in pulmonary Treg and activated Treg phenotype was found, confirming these phenotypic changes in the lung of naïve mice. Using IAV infection as a model of acute viral infection and T cell-dependent disease, this study demonstrates that the observed TRα-regulated shift translates into a significant effect on the severity of infection. Decreased body weight loss and prolonged survival, as well as reduced TH alterations, were found in TRαGS mice during low-dose infection. Inflammatory cytokines were previously shown to be critically related to disease severity, as they drive TH alterations during non-thyroidal illness, interfering with central control of TH and peripheral TH conversion (24, 25). Cytokines are also involved in body weight changes during disease related to anorexia (26, 27) and cachexia (28, 29). In line with this, reduced pro-inflammatory CD4 and CD8 T cell responses and corresponding cytokines IFN-γ and GzmB were found in TRαGS mice upon infection. Inflammation and the magnitude of CD8 T cell responses were shown to correlate with the initial infectious dose (30). Using higher doses of infection, gradual alignment of body weight loss at medium and high doses of IAV was observed in TRαGS mice, suggesting a dose-dependent effect. Thus, these findings imply that diminished cytotoxic and Th responses attenuate disease severity in TRαGS mice.

Despite prolonged survival, TRαGS mice still succumbed to infection. In line with this, no differences in tissue pathology and apoptosis were observed in TRαGS mice at the peak of infection. While tissue pathology may be related to cytotoxic immune responses in IAV-infected TRαWT mice, diminished CD8 T cell response could lead to increased viral persistence and thus tissue damage in the lungs of TRαGS mice (31, 32). The initially beneficial effect of altered TRα action may therefore change during infection.

Treg control the magnitude of pro-inflammatory CD4 and CD8 T cell responses during infection, counteracting conventional T cell activation (33). Naïve TRαGS mice showed an increased abundance of Treg in the lung and activated phenotype of Treg (6). IAV infection leads to a strong induction of Treg, again with elevated Treg frequencies in the lung of TRαGS mice at the peak of infection. These pulmonary TRαGS Treg showed an enhanced CD44^+^ CD62L^low^ effector phenotype and increased ratio of activation markers Klrg1^+^ and St2^+^ in Treg. As no differences in pro-inflammatory CD8 T cell and Th1 activation were found *in vitro* (6), enhanced activation of Treg in TRαGS mice may restrict pro-inflammatory T cell activation at the site of infection, leading to reduced disease severity. IL-10 is a common effector cytokine mediating suppressive function of Treg (34). Given the decreased concentrations of IL-10 in the lung of IAV-infected TRαGS mice, inhibitory mechanisms of TRαGS Treg seemed to be independent of IL-10. Instead, IL-10 concentrations correlated with reduced effector T cell responses in TRαGS mice, which were demonstrated to be a major source of IL-10 during IAV infection (35).

In line with the obtained results, previous studies indicate an inhibitory effect of TH on Treg immunity. Decreased conventional T cell proliferation was described during chronic stress, which is associated with reduced serum TH levels, as well as increased Treg frequencies (36, 37, 38, 39). On the contrary, TH treatment was shown to cause elevated conventional T cell numbers in a murine mammary carcinoma model (40) and to reduce Treg frequencies (41). The observed phenotype in TRαGS mice is therefore related to the lack of canonical TRα action rather than elevated serum TH concentrations detected in IAV-infected TRαGS compared to TRαWT mice. Differences in the Treg phenotype of TRαKO and TRαGS mice may be explained by opposing effects of canonical and noncanonical TRα action. Canonical TRα signaling might have an inhibitory effect on Treg, whereas noncanonical TRα action might promote Treg immunity. Thus, the absence of both pathways in TRαKO mice may have no effect, while sole abrogation of canonical signaling in TRαGS mice might lead to an enhanced Treg response. Further studies are needed to define the precise mechanisms and the role of canonical and noncanonical TRα signaling in T cells and Treg.

The absence of TRα in TRαKO mice was associated with elevated frequencies of monocytes and pro-inflammatory cytokine TNF-α during IAV infection. In previous studies, loss of TH-activating DIO2, as well as hypothyroidism, were similarly associated with increased inflammatory cytokines in a ventilator-induced lung injury (VILI) model (42). Enhanced inflammation was reversed upon T3 treatment; however, the source of inflammatory cytokines, such as innate immune cells, was not identified. During IAV infection, pro-inflammatory monocytes were demonstrated to play a detrimental role (43, 44, 45). In line with this, in the present study, increased viral loads were found in TRαKO mice upon IAV infection. These results demonstrate a limiting role of TH in acute inflammation during disease, with an essential role of TRα action in monocyte responses.

Together, these findings contribute to a better understanding of TRα signaling in immune responses and its impact on acute infections, e.g. influenza virus infection. Yet, further studies are required to finally clarify the role and mechanisms of TH/TRα signaling in Treg immunity during disease. Specifically, future studies may unravel why abrogation of only canonical TRα action is beneficial and absence of both canonical and noncanonical TRα signaling is detrimental. Cell-specific models, such as Foxp3-cre mice and Foxp3 reporter mice, may help to further decipher the cell-intrinsic impact of canonical and noncanonical TRα action on Treg and Treg suppressive function. Efficient isolation of Treg using Foxp3 reporter mice may also allow in-depth analysis of Treg function *in vitro* and *in vivo*. As the present study utilized female mice only, future studies may also elucidate the potential relevance of sex differences in the endocrine and immune systems in the given context.

**Figure 8:**
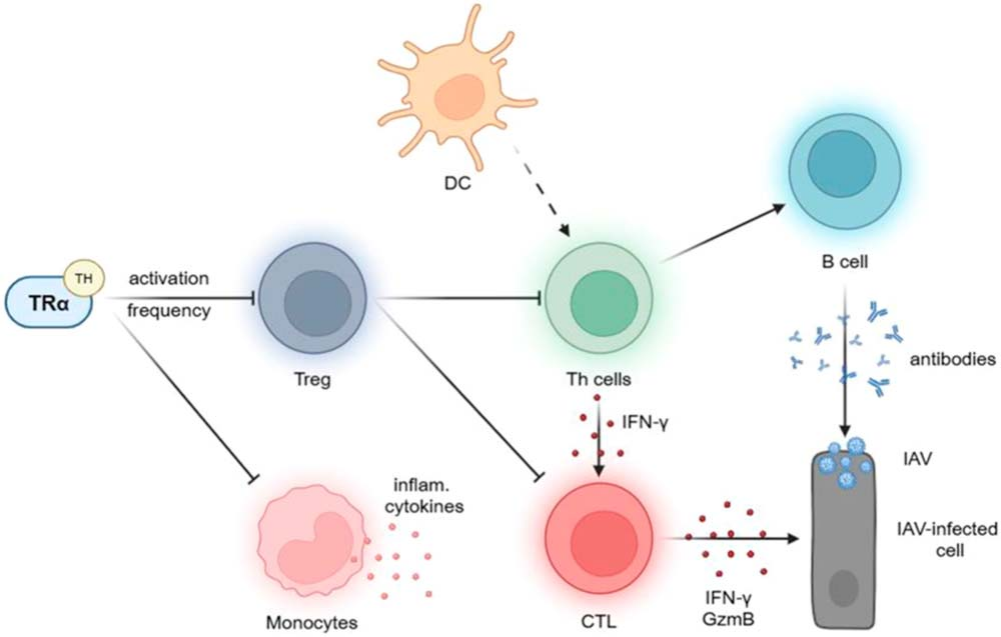
TH may regulate the frequency and activation of Treg, finally controlling pro-inflammatory, antiviral immune responses. TH, thyroid hormones; Treg, regulatory T cell; DC, dendritic cell; Th, T helper cell; CTL, cytotoxic CD8 T lymphocyte; IFN-γ, interferon-γ; GzmB, granzyme B; IAV, influenza A virus.

This study shows for the first time that TRα signaling shapes innate and adaptive immune responses during viral infection (Fig. 8). While the sole loss of canonical TRα signaling had a beneficial effect during acute IAV infection, complete loss of TRα was detrimental. These findings suggest that canonical TRα signaling controls Treg responses during infection, restricting pro-inflammatory innate and adaptive immune responses. As a result, disease severity is reduced, leading to prolonged survival. In addition, this study indicates that absence of TRα aggravates pro-inflammatory monocyte responses during disease. This differential role of TRα signaling in innate and adaptive immune cells highlights the need to implement spatio-temporal aspects of TH action to infer the therapeutic potential of TRα signaling during disease. Future studies may focus on the underlying mechanisms of local TH action to finally leverage the therapeutic potential of TH/TRα signaling in severe infections.

## Supplementary materials



## Declaration of interest

The authors declare that there is no conflict of interest that could be perceived as prejudicing the impartiality of the work reported.

## Funding

This work was supported by the Deutsche Forschungsgemeinschaft (SFB/TR 296 LOCOTACT – Project-ID 424957847, RTG 1949) and the European Thyroid Association (ETA Research Grant 2024). We acknowledge support by the Open Access Publication Fund of the University Duisburg-Essen. 

## Author contribution statement

CW: investigation (lead); writing – original draft. TK: resources; methodology; writing – review and editing. SH: conceptualization (support); methodology; writing – review and editing. AB: investigation (support); writing – review and editing. RK: investigation (support); writing – review and editing. DZ: conceptualization (support); writing – review and editing. HH: conceptualization (support); writing – review and editing. AW: conceptualization (support); resources; writing – review and editing. LM: conceptualization (support); methodology; writing – review and editing. DF: conceptualization (lead); methodology; writing – review and editing.
