# Supplementary material for: Thyroid hormone receptor α signaling shapes innate and adaptive immune responses during viral infection
Source: Eur Thyroid J. 2025 Nov 6;14(6):e250156. doi: 10.1530/ETJ-25-0156 (PMC12599294; doi:10.1530/ETJ-25-0156)
Supplement: Supplementary file 1 [file supplementary_materials.pdf]

## Supplemental Data

## Material and Methods

### In vivo IAV infection

Mice were anesthetized by ketamine (100 mg/kg body weight), xylazine (5 mg/kg body weight) and intranasally infected with low (150 PFU/ml), medium (300 PFU/ml) or high dose (600 PFU/ml) of influenza virus A/PR8/34 (50 µL). Upon infection mice were monitored daily and weight loss was recorded.

### Histopathological analysis

Lungs were perfused with PBS and filled with 4% paraformaldehyde (PFA) via the trachea. Next lungs were dissected, fixed in 4 % PFA, embedded in paraffin, sectioned at 4µm thickness and stained with hematoxylin and eosin (H&E). The severity of the histopathology was scored in a blinded manner according to the inflammation markers, including inflammatory cell infiltration, cell necrosis, epithelial hyperplasia and edema, in parenchymal, bronchial, alveolar and vascular areas of the lung. Each marker was scored from 0 (= no lesions) to 4 (= severe lesions).

To determine the number of apoptotic cells in infected lung tissue, TUNEL staining of lung sections was performed via In Situ Cell Death Detection Kit (Roche). Paraffin embedded tissue was sectioned at 3µm. Whole lung sections were imaged by Axio Scan Z1 (Zeiss) and the number of total cells (Dapi+) and TUNEL+ cells in lung tissue was quantified via ImageJ Software.

### Cytokine measurements

The concentration of cytokines was determined in serum and supernatant of lung tissues from IAV-infected mice. Lung tissues were homogenized in PBS + 0.3% BSA. Measurements were performed using Luminex Assay and Luminex MAGPIX analyzer (biotechne).

## Cell recovery

To obtain single cell suspensions of pulmonary immune cells, mice were euthanized at indicated time points. Lungs were perfused with PBS, dissected, minced and digestion of the tissue was performed at 37°C in Iscove's Modified Dulbecco's Medium (IMDM) (Invitrogen) containing 0.5 mg/mL DNase I (Roche Life Science), 0.16 mg/mL Collagenase D (Roche Life Science) and 5% heat-inactivated FBS. After 45 min the remaining tissue was passed through a 70µm nylon cell strainer and erythrocytes were lysed using ACK buffer. Finally, the cells were resolved in PBS containing 2% heat-inactivated FBS and 2 mM Ethylenediaminetetraacetic acid (EDTA) for further use.

In order to isolate murine immune cells from lymph nodes, cervical lymph nodes were dissected, passed through a 70 µm cell strainer and erythrocyte lysis by ACK buffer was performed. For further use cells were resuspended in PBS containing 2 % FBS and 2 mM EDTA.

## Thyroid hormone measurements

To measure serum TH concentrations 10µl of standards in a 6% BSA matrix, serum controls and samples were diluted with 300µl PBS and analyzed. The detection limit was 0.2nmol/L for T4 and rT3, and 0.1nmol/L for T3.

## *In vitro* proliferation assay

CD8 T cells were enriched from murine spleens using the CD8+ T cell Isolation Kit (Miltenyi Biotec). Next, cells were labelled with Vybrant™ CFDA SE Cell Tracer Kit (Invitrogen). Briefly, cells were washed with IMDM without additives. Next cells were stained in IMDM containing 2.5µM CFDA SE dye and incubated for 8 min at 37 °C. Staining was stopped adding equal amount of FBS to medium and incubating 5 min at 37 °C. For *in vitro* activation $2 \times 10^5$ labelled cells were seeded on 96-well plate (flat) and activated with 1µg/ml immobilized anti-CD3 antibody (BD Biosciences) and 1µg/ml soluble anti-CD28 antibody (BD Biosciences).

Proliferation of the cells was examined after 48h by flow cytometry based on the dilution of CFDA SE staining during cell division.

**Gene expression analysis**

An adequate amount of total RNA was reverse transcribed into cDNA with SuperScript III (18080-051, Invitrogen) and random hexamer primers. qRT-PCR was performed using PerfeCTa SYBRGreen Super Mix (95055-02K, QuantaBio) on a LightCycler LC480 (Roche). Primer sequences used are listed in Table 1. According to the MIQE guidelines for qRT-PCR, we used a set of three reference genes (GAPDH, PPIA and 18S) for accurate normalization and calculation[6]. Ct values < 35 were used for analysis and calculation of the relative change in gene expression by the efficiency-corrected method[45].

**Table 1: Primer sequences used for qRT-PCR.**

| **Sequence** | **Company** | **Accession** |
|---|---|---|
| Primer GAPDH<br>Fw-CCT CGT CCC GTA GAC AAA ATG<br>Rev-TGA AGG GGT CGT TGA TGG C | Eurofins | NM_008084.4 |
| Primer PPIA<br>Fw-CTT GGG CCG CGT CTC CTT CG<br>Rev-GCG TGT AAA GTC ACC ACC CTG GC | Eurofins | NM_013556.2 |
| Primer 18S<br>Fw-CGG CTA CCA CAT CCA AGG AA<br>Rev-GCT GGA ATT ACC GCG GCT | Eurofins | NR_003278.3 |
| Primer IAV M1<br>Fw-CTT CTA ACC GAG GTC GAA ACG<br>Rev-AGG GCA TTT TGG GAC AAA GCG TCT A | Eurofins | NC_002016.1 |

## Supplemental Figure 1

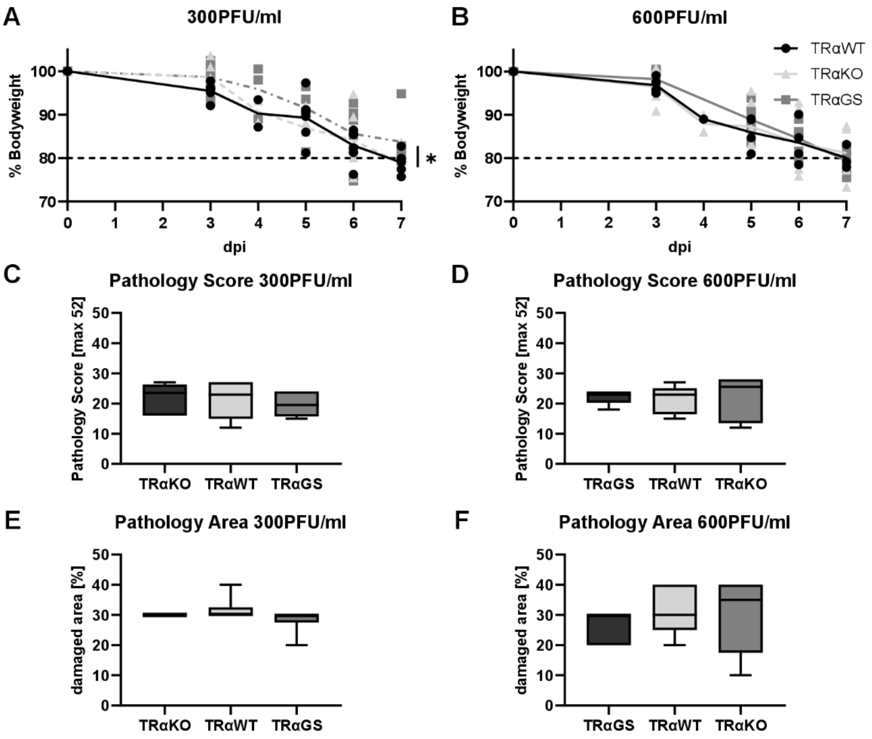


**Supplemental Fig. 1: Beneficial effects of TRαGS mice depend on the dose of infection.** (A) Body weight changes were monitored upon medium (300PFU/ml) and high dose (600PFU/ml) infection. (C-D) Histopathology was performed to score the severity of tissue damage and (E-F) the damaged area upon 300PFU/ml and 600PFU/ml infection. Data are shown as mean ± SD pooled from three independent experiments (n = 6). *p<0.05 by two-way ANOVA (A, B) or by Kruskal-Wallis test (C-F).
